# The Endocannabinoid/Cannabinoid Receptor 2 System Protects Against Cisplatin-Induced Hearing Loss

**DOI:** 10.3389/fncel.2018.00271

**Published:** 2018-08-21

**Authors:** Sumana Ghosh, Sandeep Sheth, Kelly Sheehan, Debashree Mukherjea, Asmita Dhukhwa, Vikrant Borse, Leonard P. Rybak, Vickram Ramkumar

**Affiliations:** ^1^Department of Pharmacology, Southern Illinois University School of Medicine, Springfield, IL, United States; ^2^Department of Surgery, Southern Illinois University School of Medicine, Springfield, IL, United States; ^3^Department of Otolaryngology, School of Medicine, Washington University in St. Louis, St. Louis, MO, United States

**Keywords:** endocannabinoids, CB2 receptor, cisplatin, hearing loss, ribbon synapse

## Abstract

Previous studies have demonstrated the presence of cannabinoid 2 receptor (CB2R) in the rat cochlea which was induced by cisplatin. In an organ of Corti-derived cell culture model, it was also shown that an agonist of the CB2R protected these cells against cisplatin-induced apoptosis. In the current study, we determined the distribution of CB2R in the mouse and rat cochleae and examined whether these receptors provide protection against cisplatin-induced hearing loss. In a knock-in mouse model expressing the CB2R tagged with green fluorescent protein, we show distribution of CB2R in the organ of Corti, stria vascularis, spiral ligament and spiral ganglion cells. A similar distribution of CB2R was observed in the rat cochlea using a polyclonal antibody against CB2R. *Trans*-tympanic administration of (2-methyl-1-propyl-1H-indol-3-yl)-1-naphthalenylmethanone (JWH015), a selective agonist of the CB2R, protected against cisplatin-induced hearing loss which was reversed by blockade of this receptor with 6-iodo-2-methyl-1-[2-(4-morpholinyl)ethyl]-1H-indol-3-yl](4-methoxyphenyl)methanone (AM630), an antagonist of CB2R. JWH015 also reduced the loss of outer hair cells (OHCs) in the organ of Corti, loss of inner hair cell (IHC) ribbon synapses and loss of Na^+^/K^+^-ATPase immunoreactivity in the stria vascularis. Administration of AM630 alone produced significant hearing loss (measured by auditory brainstem responses) which was not associated with loss of OHCs, but led to reductions in the levels of IHC ribbon synapses and strial Na^+^/K^+^-ATPase immunoreactivity. Furthermore, knock-down of CB2R by *trans*-tympanic administration of siRNA sensitized the cochlea to cisplatin-induced hearing loss at the low and middle frequencies. Hearing loss induced by cisplatin and AM630 in the rat was associated with increased expression of genes for oxidative stress and inflammatory proteins in the rat cochlea. *In vitro* studies indicate that JWH015 did not alter cisplatin-induced killing of cancer cells suggesting this agent could be safely used during cisplatin chemotherapy. These data unmask a protective role of the cochlear endocannabinoid/CB2R system which appears tonically active under normal conditions to preserve normal hearing. However, an exogenous agonist is needed to boost the activity of endocannabinoid/CB2R system for protection against a more traumatic cochlear insult, as observed with cisplatin administration.

## Introduction

Cisplatin is one of the main chemotherapeutic agents used to treat different solid tumors including head and neck cancer, testicular and ovarian cancer. However, cisplatin chemotherapy results in dose-limiting toxicity to the cochlea, kidney, and peripheral neurons. Hearing loss derives from the generation of significant oxidative stress in the cochlea which results in part from the activation and induction of a cochlear-specific NADPH oxidase isoform, NOX3 (Banfi et al., 2004). The oxidative stress coupled to the subsequent inflammatory processes and DNA damage occurring in the cochlea promotes apoptosis and necrosis of the sensory outer hair cells (OHCs) at the basal and middle regions of cochlea (Estrem et al., 1981; Laurell and Bagger-Sjoback, 1991; van den Berg et al., 2006; Rybak et al., 2007), damage to the stria vascularis (SV) (Meech et al., 1998) and loss of spiral ganglion neurons (SG) (Zheng et al., 1995; van Ruijven et al., 2005). Currently, there are no drugs which are currently approved by the US FDA for treating cisplatin-mediated hearing loss (Rybak et al., 2009). We believe that a better understanding of the mechanisms involved in ototoxicity and endogenous protective signaling pathways could stimulate the development of effective and novel otoprotective drugs.

Cannabinoid (CB) receptors include cannabinoid receptor 1 (CB1R), cannabinoid receptor 2 (CB2R), non-CB1/CB2Rs, such as transient potential vanilloid 1 receptor (TRPV1), and G protein-coupled receptor 55 (GPR55) (Console-Bram et al., 2012). High levels of CB1R are expressed in the central nervous system (CNS), while CB2R are predominantly expressed in immune cells of the body including macrophages, leucocytes, dendritic cells and natural killer cells (Console-Bram et al., 2012). CB2R regulate the release of pro-inflammatory cytokines such as interleukins (IL-1β, Il-6), tumor necrosis factor-α (TNF-α), cyclooxygenase-2 (COX-2) and chemokines (CXCL8, CCL2) from leucocytes and the differentiation of B and T cells (Klein, 2005). CBs also suppress the activity of helper T cells (Th1), increase the activity of Th2 cells (Klein, 2005) and reduce leucocyte recruitment to the site of inflammation (Klein, 2005; Pacher et al., 2006). Activation of CB2R regulates the cell growth and proliferation of immune cells and suppresses macrophage growth and phagocytosis (Ashton and Glass, 2007). Activation of endogenous cannabinoid receptors protects against inflammatory diseases such as colonic inflammation in colitis (Massa et al., 2004), rheumatoid arthritis (Zurier et al., 2003) and autoimmune diabetes (Weiss et al., 2008). Endogenous ligands for CB receptors, termed endocannabinoids, comprise anandamide and 2-arachidonoyl glycerol (2-AG).

Previous studies have documented expression of CB2R in immortalized House Ear Institute - Organ of Corti cell line (HEI-OC1) which protected against cisplatin-induced cell death (Jeong et al., 2007). A recent report showed that CB2Rs are expressed in the rat cochlea and are induced by cisplatin treatment (Martin-Saldana et al., 2016). However, no functional role of these receptors was demonstrated. In other disease models, upregulation of CB2R has been shown to mediate an “autoprotective” role (Pertwee, 2009). Increased expression of CB2R was observed in neuropathic pain models (Zhang et al., 2003) and in rat sensory neurons during peripheral nerve injury (Wotherspoon et al., 2005).

In the current study, we provide evidence that CB2R plays a central role in regulating normal hearing, as inhibition or knockdown of this receptor results in hearing loss. Furthermore, *trans*-tympanic application of an agonist of CB2R protected against cisplatin-induced hearing loss. These findings support the conclusion that drugs targeting the CB2R could boost the endocannabinoid system and play an important role in protecting against hearing loss.

## Materials and Methods

### Drugs and Reagents

CB2 agonist, (2-methyl-1-propyl-1H-indol-3-yl)-1-naphthalenylmethanone (JWH015), and antagonist, 6-Iodo-2-methyl-1-[2-(4-morpholinyl)ethyl]-1H-indol-3-yl](4-methoxyphenyl)methanone (AM630), were purchased from Tocris (#1341 and #1120, respectively). The different antibodies used for this project were purchased from different vendors and are as follows: CB2R (#Ab45942) and CB2R blocking peptide (#Ab45941) from Abcam, antibody for Tuj1 was obtained from Covance (#MMS-435P), while anti-CtBP2 was obtained from BD Biosciences (#612044) and Anti-GluR2 was obtained from Millipore (#MAB397). Antibody against Na^+^/K^+^-ATPase-α1 was obtained from Santa Cruz Biotechnology (#sc21712) while Alexa Fluor^TM^ 488 Phalloidin antibody was obtained from Thermo Fisher Scientific (#A12379). Two different myosin VIIa antibodies were used for different purposes: Mouse IgG1 anti-myosin-VIIa antibody was obtained from Developmental Studies Hybridoma Bank (#138-1), while rabbit anti-myosin-VIIa was purchased from Proteus Biosciences (#25-6790). Alexa Fluor 647 goat anti-rabbit antibody (#A21244), Alexa Fluor 488 goat anti-rabbit antibody (#A11008), Alexa Fluor 647 goat anti-mouse IgG1 antibody (#A21240), Alexa Fluor 568 goat anti-mouse IgG2a antibody (#A21134) and Alexa 568 goat anti-mouse IgG1 antibody (#A21124) were obtained from Life Technologies. Donkey anti-rabbit IRDye 680RD (#926-68073) and goat anti-mouse IRDye 800RD (#926-32214) were from LI-COR biosciences. Detailed description of the primary antibodies used is provided in the **Supplementary Table S1**. For culturing cells, RPMI1640 media was ordered from Gibco Life Technologies (#11875085).

### Animal Procedures

All the animals were housed in the Division of Laboratory Animal Medicine (DLAM) facility of SIU School of Medicine and they were provided with easy access to commercial food and water. The rooms had controlled temperature and normal light/dark cycles (12 h light/12 h dark). All experiments performed on animals were approved and monitored by the Southern Illinois University School of Medicine Laboratory Animal Care and Use Committee.

Mice expressing the CB2R tagged with green fluorescent protein (GFP) were kindly provided by Dr. Cecilia J. Hillard (Department of Pharmacology and Toxicology, Medical College of Wisconsin). The mice (5–6 weeks old) were anesthetized using isoflurane and sacrificed. Different organs including cochlea, spleen and cerebellum were isolated and fixed in 4% paraformaldehyde for 4–6 h in 4°C, followed by washing in 10 mM PBS and stored at 4°C until use.

Adult male Wistar rats (weight 200–250 g, 2–3 months old) were purchased from Envigo (Indianapolis, IN, United States). Rats were anesthetized with a mixture of ketamine (90 mg/kg) and xylazine (17 mg/kg) and the depth of anesthesia was determined by the absence of reflex to toe pinch which was performed routinely during the duration of the procedure. If the depth of anesthesis was determined to be insufficient, the rats were administered additional dose of the mixture. Auditory brainstem responses (ABRs) were performed on anesthetized rats in a radio frequency concealed sound chamber just prior to *trans*-tympanic JWH015 or AM630 treatments (for experimental group) or PBS of pH 7.2 (for control groups). Cisplatin (11 mg/kg) was administered via the intraperitoneal route and post-treatment ABRs were measured 72 h later. At this point, the animals were anesthetized using the same procedure mentioned before and decapitated. One cochlea from each animal was fixed using cochlear perfusion of 4% paraformaldehyde for immunohistochemistry studies and the other cochlea was snap-frozen for RNA extraction.

### Auditory Brainstem Responses

Auditory evoked potentials were recorded as described previously (Sheehan et al., 2018). Briefly the stainless steel electrodes were positioned as follows: the ground electrode in the rear flank, the positive electrode between the two ears directly atop the skull, and the negative electrodes below the pinna of each ear. Acoustic stimuli were delivered through inserted earphones as a 5 ms tone burst at 8, 16, and 32 kHz and stimulus intensities were determined as decibel sound pressure level (dB SPL) which began at 10 dB SPL and reached ultimately 90 dB SPL with a 10 dB step size. Threshold was defined as the lowest sound intensity capable of evoking a reproducible, visually detectable response with two distinct waveforms with minimum amplitudes (II and III) of 0.5 μV. Threshold shift represents the difference in threshold measured after treatment compared with threshold obtained prior to treatment. Wave I was identified from the neural responses and wave I amplitudes were documented from the ABRs obtained from the different treatment groups.

### *Trans*-Tympanic Administration of Drugs and siRNA

Rats were anesthetized with ketamine/xylazine mixture. A single *trans*-tympanic injection of the drug or siRNA was made in the anterior–inferior region of rat tympanic membrane using a 28–30 gauge needle (Sheehan et al., 2018). The position of the needle was visualized using a Zeiss operating microscope and 50 μl of solution was injected into the middle ear (siRNA was resuspended in 50 μl of sterile water to get the desired concentration). A similar procedure was performed 30 min later on the other ear.

### siRNA Sequences

The rat CB2R siRNA was purchased from Dharmacon as an ON-TARGETplus SMARTpool siRNA which includes a combination of four siRNA sequences against CB2R RNA. The target sequence of CB2R siRNA included in this combination are as follows: 5′-GCUUGGAGUUCAACCCUAU-3′; 5′-CCAAUCAGCCUCCCUAAUA-3′; 5′-CUUGUUAACUCCAUGAUCA-3′ and 5′-UGACCGCUGUUGACCGAUA-3′. ON-TARGETplus Non-targeting Control siRNAs (scramble), also purchased from Dharmacon, was used as negative control.

### Cochlear Whole-Mount Preparation

Isolated mice and rat cochleae were perfused with 4% paraformaldehyde and kept overnight at 4°C in the same solution for fixation. Cochleae were then decalcified in 100 mM EDTA (pH 7.3) with stirring at room temperature for 3 weeks for rat cochlea and 4 days for mouse cochlea, respectively. The decalcified cochleae were then microdissected into basal, middle and apical turns for immunolabeling.

### Immunohistochemistry

Decalcified cochleae were dissected and whole mount sections were blocked, permeabilized in a mixture of 1% Triton X-100, 1% BSA and 10% horse or goat serum depending on the host species used for primary antibodies at room temperature for 1 h. They were then incubated with primary antibodies diluted in a solution containing 0.1% Triton X-100, 1% BSA and 10% horse or goat serum. All primary antibodies were incubated at 4°C overnight on a rotator. The cochleae were then washed three times and then placed in secondary antibodies, washed, mounted on slides and used for imaging with Zeiss LSM800 scanning confocal microscope. Mid-modiolar sections (10 μm thickness) of fixed and decalcified cochlea were obtained by using a cryostat, which were then dehydrated by incubating in 100% ethanol at −20°C for 20 min, followed by rehydration. These sections were stained as above. Fluorescence intensity of the staining was quantified using ImageJ software.

### Hair Cell and Ribbon Synapse Count

The whole mount preparations of the cochlea samples were labeled with antibodies against myosin VIIa, CtBP2, and GluR2 for labeling of hair cells, presynaptic RIBEYE and post-synaptic glutamate receptors, respectively. The average hair cell counts in each region were determined by manually counting cells in the basal turn (100 μm length) of the cochlea. Normal counting yield was 39–42 OHCs per 100 μm. The average number of OHCs per three such regions taken at random from the base and averaged to give the mean OHC count per animal per treatment group. Functional ribbon synapses were defined as the number of complexes showing both CtBP2 + GluR2 immunolabeling apposing each IHC. At least 30 IHCs were used per sample to provide an estimate of functional synapses per rat cochlea. At least 3–4 animal cochleae were counted per group and used for statistical comparisons.

### RNA Isolation and Real-Time RT-PCR

Total RNA was isolated from the cochlea as described previously (Mukherjea et al., 2011). The purity and concentration of RNA was determined by measuring the optical densities at 260, 280, and 230 nm by a nanodrop spectrophotometer. The purified RNA sample (260/230 ratios ranged from 1.8 to 2.0) were used for PCR. PCR was performed using 500 ng of total RNA for gene specific cDNA synthesis by using iScript Select cDNA Synthesis Kit (Bio-Rad). The procedure used for real-time RT-PCR was described previously (Mukherjea et al., 2011). The cycle threshold (*C*t) was determined by the number of cycles at which the samples reached threshold fluorescent intensity. Negative controls were set up for both the target and housekeeping gene, GAPDH, for all reaction groups and no template cDNA was added to this mixture. The relative change in mRNA levels between the vehicle and treated group was quantified by the following formula: 2^ΔΔC_t_^ (Soong et al., 2001). The nucleotide sequences of rodent primer sets were based on homologous sequences of rat and mouse cDNA sequence. The primers were purchased from Sigma Genosys (St. Louis, MO, United States) and the primer sets used are listed below:

Rodent-GAPDH (sense): 5′-ATGGTGAAGGTCGGTGTGAAC-3′ (antisense): 5-TGTAGTTGAGGTCAATGAAGG-3′Rodent-TRPV1 (sense): 5′- CAAGGCTGTCTTCATCATCC-3′ (antisense): 5-AGTCCAGTTTACCTCG TCCA-3′ Rodent-NOX3 (sense): 5′ GTGAACAAGGGAAGGCTCAT-3′ (antisense): 5′-GACCCACAGAAGAACACGC-3′ Rodent-TNF-α (sense): 5′-CAGACCCTCACACTCAGATCA-3′ (antisense): 5′-TGAAGAGAACCTGGGAGTAGA-3′ Rodent-COX-2 (sense): 5′-TGATCGAAGACTACGTGCAAC-3′ (antisense): 5′-GTACTCCTGGTCTTCAATGTT-3′ and Rodent-iNOS (sense): 5′-CATTCTACTACTACCAGATC-3′ (antisense): 5′-ATGTGCTTGTCACCACCAG-3′ and Rodent KIM-1 (sense): 5′-TTCAAGTCTTCATTTCAGGCC-3′(antisense): 5′-CTGCTCCGATAGGTGACTTGG-3′.

### Cell Cultures

Immortalized mouse organ of Corti cells UB/OC-1 cells were kindly provided by Dr. Matthew Holley (Institute of Molecular Physiology, Western Bank, Sheffield, United Kingdom). Cells were cultured in RPMI-1640 media supplemented with 10% Fetalclone II serum (Hyclone), 5% penicillin–streptomycin (Invitrogen) and normocin (InvivoGen). Cultures were grown at 33°C in a humidified incubator with 10% CO_2_. Head and neck cancer cell line [University of Michigan squamous carcinoma cells (UMSCC)10B], ovarian cancer cell (HeyA8) and colon cancer cells (HCT116 WT) were cultured in RPMI-1640 media supplemented with 10% FBS (Atlanta Biologicals) and 5% penicillin-streptomycin at 37°C and 5% CO_2_. Confluent monolayer cells were used for experiments and cells were cultured thrice a week for passaging.

### Plasmid Transfection in UB/OC-1 Cells

Wild type CB2 receptor expressing pcDNA3.1 plasmids were purchased from cDNA resource center. *E. coli* bacteria were transformed with this plasmid and the transformed colonies were selected by ampicillin resistance. DNA was isolated from the transformed bacteria by maxi-prep (Qiagen) and transfected into UB/OC1 cells by using Lipofectamine 3000 reagent (Invitrogen) following the vendor’s protocol.

### Immunocytochemistry

To detect the expression of CB2R in cells, UB/OC-1 cells were plated in 24 well dishes on coverslips in complete media. The confluent monolayer of cells was washed three times with ice-cold 1X PBS and fixed with 4% paraformaldehyde for 15–20 min at room temperature. The staining procedure was the same as mentioned above for immunohistochemistry. The slides were imaged by Zeiss LSM800 scanning confocal microscope.

### MTS Assay for Cell Viability

*In vitro* cell viability of cancer cells following different drug treatments was measured by using CellTiter 96^®^ Aqueous One Solution Cell Proliferation Assay kit (Promega). HeyA8 (2,000 cells per well), UMSCC10B and HCT116 WT (2,500 cells per well) cells were plated in 96 well plate. The cells were treated with JWH015 (10 μM) for 30 min followed by cisplatin (10 μM) for 48 h. At the end point, 20 μl of Cell Titer Aqueous One solution reagent was added to each well containing 100 μl media. The cells were incubated for at least 45 min in 33°C and checked for any color development and the plates were read at a wavelength of 490 nm by Fluoroskan Ascent^TM^ FL Microplate Fluorometer plate reader. For each cell line, experiments were repeated independently at least three times and averages from independent repeats were used for statistical analyses. The percentage of cell viability was normalized against vehicle treated cells.

### Statistical Analyses

The statistical significance differences were evaluated by using either student’s *t*-test for two groups or Analysis of Variance (ANOVA) with Tukey’s *post hoc* test for multiple treatment groups using Graph Pad Prism software 6.0.

## Results

### CB2 Receptors Are Expressed in the Mouse and Rat Cochlea

**FIGURE 1:**
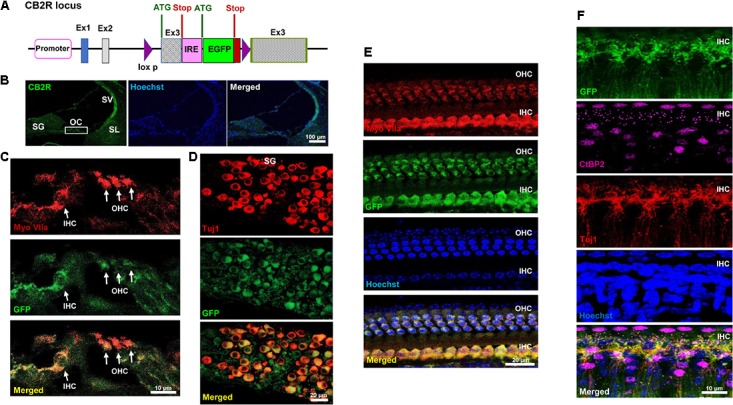
Distribution of the GFP-tagged CB2R knock-in mice. **(A)** The genetic background of the knock-in mice is shown where the GFP reporter gene was inserted within exon 3 of CB2R and the expression of GFP was driven by internal ribosomal entry (IRE) promoter. Cochleae isolated from these GFP-CB2R co-expressing knock-in mice were fixed in 4% paraformaldehyde followed by decalcification and then sectioned. **(B)** The representative mid-modiolar section shows endogenous GFP fluorescence (green) in different anatomical regions of the cochlea including organ of Corti (OC), stria vascularis (SV), spiral ligament (SL), and spiral ganglion (SG) neuron. **(C)** Immunolabeling of GFP-tagged CB2R (green) along with hair cell marker, Myo VIIa (red), show colocalization of CB2R in both, the OHCs and IHCs. **(D)** Co-labeling of these cochleae with Tuj1 (red), a neuronal marker, show the distribution of GFP-CB2R (green) in spiral ganglion neurons. **(E)** Whole mount sections of cochleae from GFP-CB2R co-expressing knock-in mice show co-localization of GFP-CB2R (green) and myosin VIIa (red) in the OHCs and IHCs of OC. **(F)** GFP-CB2R whole mounts sections were also co-stained with neuronal marker, Tuj1 (red), and pre-synaptic ribbon marker, CtBP2 (magenta), showing pre-synaptic localization of CB2R. Cell nuclei (blue) are stained with Hoechst stain in **(B,E,F)**. Studies reported here were repeated in at least three different animals and similar results were obtained.

CB2R immunolabeling in the rat cochlea has been reported previously using commercially available CB2R antibody (Martin-Saldana et al., 2016). However, the specificity of this antibody is controversial (Baek et al., 2013). We therefore validated the distribution of CB2R in the cochlea using a GFP-tagged CB2R conditional knock-in mouse model using a commercially available antibody. In the knock-in mice model, GFP was inserted within exon 3 of the CB2R, and the expression of GFP was driven by the endogenous CB2R promoter (**Figure 1A**). In mid-modiolar sections of cochleae obtained from these mice, we show endogenous GFP fluorescence in the organ of Corti (OC), spiral ganglion (SG) neurons, stria vascularis (SV), and spiral ligament (SL) (**Figure 1B**). We demonstrate the expression of GFP-CB2R in the three rows of OHC and inner hair cells (IHC) in mid-modiolar sections using co-immunolabeling of hair cells with myosin VIIa, a hair cell marker (**Figure 1C**). To demonstrate the distribution of CB2R in cochlear neurons we used Tuj1, a mouse monoclonal antibody for class III β-tubulin which labels both Type I and Type II SG neurons in the rodent cochlea (Flores-Otero et al., 2007; Lallemend et al., 2007; Barclay et al., 2011). Immunolabeling with Tuj1 showed co-localization of GFP-CB2R with Tuj1 in the soma or cell bodies (**Figure 1D**) and neurites (**Figure 1F**) of SG neurons. Whole mount preparations show co-localization of GFP-CB2R and myosin VIIa in the organ of Corti (**Figure 1E**). Presynaptic localization of CB2R has previously been shown in the autaptic hippocampal neurons (Atwood et al., 2012). Therefore, we investigated the synaptic localization of CB2R in the cochlea. Labeling with CtBP2, a presynaptic marker for the IHCs, we show co-localization of endogenous GFP-tagged CB2R and CtBP2 (**Figure 1F**).

**FIGURE 2:**
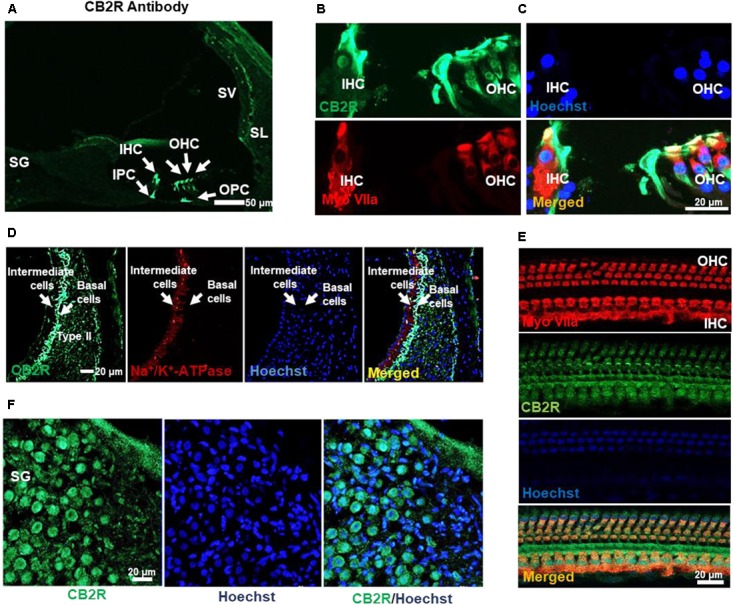
Distribution of the CB2R in the rat cochlea. **(A)** Immunolabeling of mid-modiolar cochlear sections with CB2R antibody shows labeling of cells in the organ of Corti (green). Labeling was observed in the outer hair cells (OHC), inner hair cells (IHC), inner pillar cell (IPC), and outer pillar cell (OPC). **(B)** Co-labeling of the organ of Corti with antibodies for CB2R (green) and myosin VIIa (red) showed co-localization of these proteins in the OHC and IHC. **(C)** Staining of CB2R was also observed in the intermediate and basal cells of the SV and type II fibrocytes in SL. CB2R (green) showed some overlap with Na^+^/K^+^-ATPase α1 subunit (red) in the SV. **(D)** CB2R was localized to the plasma membranes of SG. Approximately 60% of cells in this section were stained with both CB2R (green) and Hoechst (blue). **(E)** Co-labeling of CB2R and myosin VIIa in OHCs and IHCs in whole mount preparations of the organ of Corti shows that distribution of CB2R (green) is more widespread than that of myosin VIIa (red). Hoechst was used as a nuclear stain (blue) in **(B–E)**. Studies reported here were repeated in at least three different animals and similar results were obtained.

CB2R from adult male Wistar rat cochleae were characterized using a polyclonal antibody raised against amino acid residues 200–300 of this protein. This antibody was previously characterized using blocking peptide controls, inclusion of which resulted in no discernable signals in immunolabeling studies (**Supplementary Figure S1A**). Using this antibody, we observed high levels of immunolabeling in the rat spleen (which is rich in CB2R), while low levels were detected in the hippocampus (which normally show low levels of CB2R) (data not shown). In addition, higher levels of CB2R were detected in Chinese hamster ovary (CHO) cells which were transiently transfected with the CB2R expression plasmid, as compared to cells transfected with the empty plasmid vector alone (data not shown). Increased CB2R immunoreactivity was also demonstrated in organ of Corti-derived UB/OC-1 cells which were transiently transfected with the CB2R plasmid (**Supplementary Figure S1B**). Using this antibody for immunolabeling of rat cochlear sections, we showed CB2R immunoreactivity in both the OHCs and IHCs, inner (IPC) and outer pillar cells (OPC) (**Figures 2A,B**) and SG neurons (**Figure 2D**). Mid-modiolar sections were co-labeled with antibodies against Na^+^/K^+^-ATPase α1 subunit and CB2R to investigate the expression of this receptor in the SV. Intense CB2R immunolabelling was observed in the basal cells of SV, as compared to the intermediate and marginal cells (**Figure 2C**). CB2Rs were also distributed in Type II fibrocytes of the SL (**Figure 2C**). Co-labeling of CB2R and myosin VIIa was also detected in OHCs and IHCs of cochlear whole mount preparations prepared from the rat cochleae (**Figure 2E**).

### *Trans*-Tympanic Administration of CB2R Agonist Attenuated Cisplatin-Mediated Hearing Loss and Loss of OHCs

**FIGURE 3:**
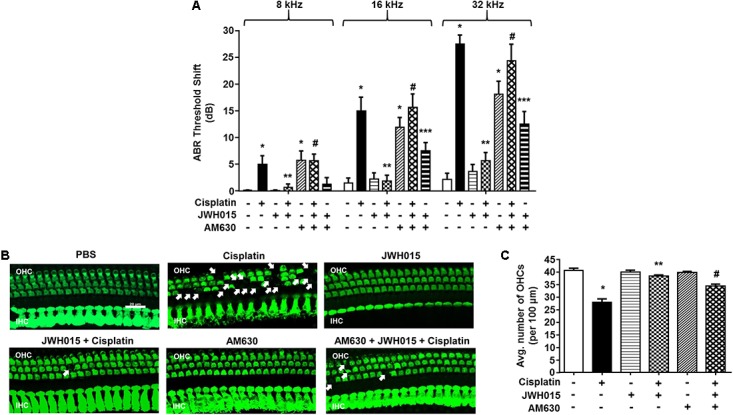
*Trans*-tympanic administration of JWH015 prevents cisplatin-induced hearing loss. **(A)** Baseline ABR thresholds were recorded in Wistar rats, which were then pre-treated with either *trans*-tympanic AM630 (2.5 nmoles) for 30 min followed by *trans*-tympanic JWH015 (2.5 nmoles) or individually with *trans*-tympanic AM630 or JWH015 for 24 h. Cisplatin (11 mg/kg) was then administered (i.p.) after day 1 and post-ABR was performed after 72 h. Pre-treatment with JWH015 diminished cisplatin-induce ABR threshold shift in all the three frequency regions. The protective action of JWH015 was completely blocked by AM630. *Trans*-tympanic administration of AM630 alone significantly elevated by ABR thresholds. Data (threshold shift) is plotted as mean ± SEM (*n* = 12). **(B)** The animals were sacrificed at the end point and cochleae were collected, fixed by 4% PFA in PBS followed by decalcification for 3 weeks in 100 mM EDTA. The base of the cochlea were dissected out and stained with hair cell marker, myosin VIIa, to determine the hair cell loss. Representative images are shown. Scale bar is 20 μm. **(C)** The bar graph represents the average number of hair cells per 100 μm area shown in **(B)** (*n* ≥ 3). Pre-treatment with JWH015 reduced cisplatin-induced outer hair cell loss. Administration of AM630 produced no hair cell loss. ^∗^*p* < 0.05 vs. vehicle; ^∗∗^*p* < 0.05 vs. cisplatin; ^#^*p* < 0.05 vs. JWH015 + cisplatin; and ^∗∗∗^*p* < 0.05 vs. JWH015 (one-way ANOVA).

Male Wistar rats were administered vehicle or JWH015 (2.5 nmoles in PBS per ear) by *trans*-tympanic injections, followed 30 min later by intraperitoneal cisplatin (11 mg/kg) administration. This dose of JWH015 produced optimal otoprotection than lower doses of JWH015 tested and is calculated to achieve a low micromolar range in the perilymph, assuming a 1–10% entry of the drug from the middle ear to the perilymph. Administration of vehicle produced no change in ABR thresholds when compared to pre-treatment ABRs (**Figure 3A**), attesting to the efficiency of the *trans*-tympanic drug delivery. Cisplatin increased ABR thresholds by 5.0 ± 1.5, 15.0 ± 2.5, and 27.0 ± 1.7 dB at 8, 16, and 32 kHz, respectively. Pretreatment with JWH015 diminished ABR threshold shifts in all the three frequency regions. The ABR threshold shifts were 0.6 ± 0.6, 1.9 ± 1.0, and 5.6 ± 1.5 dB at 8, 16, and 32 kHz, respectively, for the JWH015 + cisplatin treatment group. This protective action was completely blocked by *trans*-tympanic administration of AM630 (2.5 nmoles in PBS per ear), an antagonist of the CB2R (Morgan et al., 2009). ABR threshold shifts induced by cisplatin in rats pretreated with AM630 + JWH015 were 5.6 ± 1.2, 15.6 ± 2.4, and 24.3 ± 3.1 dB at 8, 16, and 32 kHz, respectively, which were similar to those obtained with cisplatin alone. An unexpected finding was that *trans*-tympanic administration of AM630 alone significantly elevated ABR thresholds by 5.8 ± 1.7, 11.9 ± 1.8, and 18.1 ± 2.4 dB at 8, 16, and 32 kHz, respectively, compared to vehicle-treated rats. Cisplatin-induced hearing loss was associated with a reduction in OHC number, assessed at the basal turn (**Figures 3B,C**). This loss of OHCs was reduced by *trans*-tympanic JWH015 administration. Administration of AM630 alone did not produce any loss of OHCs nor did it enhance the cell loss induced by cisplatin (**Supplementary Figure S2**). This finding suggests that endogenous activation of the cochlear CB2R (by endocannabinoids) helps in maintaining normal hearing and that blockade of this tonic stimulation results in hearing loss. Moreover, exogenous CB2 ligand can prevent cisplatin-induced hearing loss.

### CB2R Agonist Protected Against Cisplatin-Induced Loss of IHC Ribbon Synapse

**FIGURE 4:**
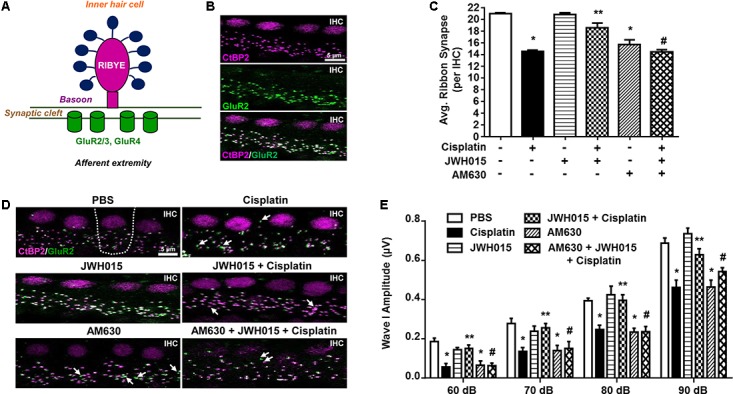
Activation of CB2R protects against cisplatin induced loss of ribbon synapse. **(A)** The cartoon represents a typical ribbon synapse at the inner hair cell (IHC). It is primarily composed of two major components: presynaptic RIBEYE and glutamate receptors (GluR) at the post-synapse. **(B)** Representative images shows whole mount preparations co-stained with presynaptic CtBP2 (for RIBEYE, magenta) and postsynaptic GluR2 (green). Functional ribbon synapses are complexes showing both CtBP2 and GluR2 immunolabeling (white) apposing each IHC. **(D)** The whole mount preparation form the basal region of cochleae from treated animals were dissected and stained for CtBP2 and GluR2. Representative images are shown. White arrows indicate single staining of either CtBP2 or GluR2. Functional synapse complexes (CtBP2 + GluR2) were quantified by averaging the numbers from 30 IHCs per samples. The bar graph **(C)** represents the average number of synaptic complexes (mean ± SEM) per IHC (*n* ≥ 4). Pre-treatment with JWH015 protected against cisplatin-induced loss of functional synapse. CB2R antagonist, AM630, administered alone or in combination with JWH015 + cisplatin produced significant reductions in functional ribbon synapses. **(E)** ABR wave I amplitude of different treatment groups at 60, 70, 80, and 90 dB SPL (32 kHz) was computed from sorted ABR wave forms. The bar graph represents mean wave I amplitude ± SEM (*n* = 12). Cisplatin decreases wave I amplitude indicating synaptopathy which was completely reversed by pre-treatment with JWH015. Blockade of CB2R activation by AM630 resulted in decrease in wave I amplitude. ^∗^*p* < 0.05 vs. vehicle; ^∗∗^*p* < 0.05 vs. cisplatin; and ^#^*p* < 0.05 vs. JWH015 + cisplatin (one-way ANOVA).

Significant loss of ribbon synapses has been reported following exposure to loud noise or treatment with aminoglycosides (Kujawa and Liberman, 2009; Chen et al., 2012; Zhang et al., 2013). Loss of ribbon synapses following noise exposure is mediated by glutamate (Puel et al., 1998). A functional ribbon synapse complex comprises a presynaptic ribbon containing RIBEYE and post-synaptic glutamate receptors, such as GluR2 (**Figure 4A**). RIBEYE can be labeled by antibodies against C-terminal binding protein 2 (CtBP2). In a recent report, we demonstrated that CtBP2 immunoreactivity was decreased following cisplatin treatment in rats and that this was abrogated by pretreatment with EGCG (Borse et al., 2017). Here we show that CB2R could also protect against cisplatin-induced loss of ribbon synapses. Functional ribbon synapses were identified as co-localization of both CtBP2 and GluR2 (**Figure 4B**). We show a significant loss of functional ribbon synapse in the basal turn from 20.9 ± 0.1 per IHC to 14.5 ± 0.2 per IHC, following cisplatin treatment (**Figures 4C,D**). Pre-treatment with JWH015 by *trans*-tympanic injection significantly reduced cisplatin-mediated loss of functional synapses. The mean number of functional synapses in JWH015 + cisplatin treated animal was 18.5 ± 0.8. This finding suggests that CB2R protects both the sensory hair cells and ribbon synapses, at least in the basal turn of the cochlea, which could account for its overall efficacy in reducing cisplatin ototoxicity. AM630 administered alone or in combination with JWH015 + cisplatin produced significant reductions in the number of ribbon synapses (15.6 ± 0.8 and 14.4 ± 0.4, respectively), compared to vehicle controls (**Figures 4C,D**). These data suggest that synaptic ribbons are a potential target of AM630 for mediating hearing loss, as this drug did not produce any loss in OHCs. Thus, it appears that one of the functions of endogenous cannabinoids in the cochlea is to maintain the integrity of ribbon synapses which could contribute significantly toward preserving normal hearing.

Synapse loss after acoustic trauma results in reductions in supra-threshold amplitudes of wave I of the ABR (Bharadwaj and Shinn-Cunningham, 2014). This phenomenon is considered to be a good indicator of cochlear synaptopathy (Kujawa and Liberman, 2009). Therefore, we determined whether cisplatin-mediated cochlear synaptopathy is associated with change of wave I supra-threshold amplitude. ABR wave I amplitude elicited by 60–90 dB SPL tones delivered at 32 kHz were significantly reduced by 33% (from 0.69 ± 0.03 μV to 0.46 ± 0.04 μV) following cisplatin treatment (*p* < 0.0001, *n* = 12) (**Figure 4E**). These decreases in wave I amplitudes were attenuated in rats pretreated with *trans*-tympanic JWH015 prior to administration of cisplatin (mean amplitude of wave I was 0.63 ± 0.03 μV), suggesting that activation of CB2R in the cochlea regulates cisplatin-induced cochlear loss of wave I amplitude. Rats treated with AM630 alone or in combination of JWH015 + cisplatin showed significantly diminished wave I amplitudes (mean amplitude 0.46 ± 0.03 μV), compared to the control animals, suggesting that CB2R activation provides tonic maintenance of normal cochlear physiology.

### Anti-inflammatory Role of CB2R in the Cochlea

**FIGURE 5:**
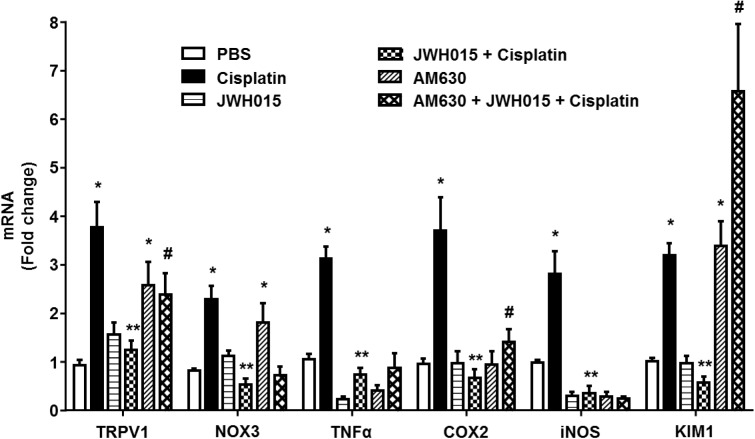
Activation of CB2R by JWH015 mitigates cisplatin-induced inflammation in the rat cochlea. Snap frozen cochlea from treated animals were used for total RNA extraction which was then converted to cDNA and used for real-time RT-PCR for the expression of *TRPV1*, *NOX3*, *TNF-α*, *COX2*, *iNOS*, and *KIM1.* Housekeeping gene, *GAPDH*, was used for normalization. Cisplatin significantly increased the expression of all the genes tested. The expression of these genes were significantly reduced in rats pre-treated with JWH015 followed by cisplatin. Administration of CB2R antagonist, AM630, alone increased the expression of *TRPV1*, *NOX3*, and *KIM1*. Data is plotted as mean fold change ± SEM (*n* ≥ 5). ^∗^*p* < 0.05 vs. vehicle; ^∗∗^*p* < 0.05 vs. cisplatin; and ^#^*p* < 0.05 vs. JWH015 + cisplatin (one-way ANOVA).

CB2Rs are primarily distributed in the immune cells where they play a key role in the regulating both humoral and innate immunity (Klein et al., 1998). These receptors suppress the growth of macrophages and phagocytes and modulate the release of inflammatory cytokines from leukocytes by regulating the release of pro- and anti-inflammatory cytokines from helper T cells, Th1 and Th2, respectively (Ashton and Glass, 2007). Inflammation plays a critical role in cisplatin ototoxicity which involves induction of pro-inflammatory cytokines by STAT1 (Kaur et al., 2011, 2016). Cisplatin increased the expression of STAT1-regulated genes, such as *TRPV1*, *NOX3*, *TNF-α*, *COX2*, *iNOS*, and *KIM1* by 3.8 ± 0.5-, 2.3 ± 0.3-, 3.1 ± 0.3-, 3.7 ± 0.7-, 2.8 ± 0.5-, and 3.2 ± 0.2-fold, respectively, compared to vehicle treated animals (**Figure 5**). These genes were significantly reduced in rats pre-treated with JWH015 followed by cisplatin. Interestingly, application of AM630 alone led to increased expression of *TRPV1*, *NOX3*, and *KIM1* by 2.6 ± 0.5, 1.8 ± 0.4 and 3.4 ± 0.1-fold, respectively, possibly indicating a functional role of endocannabinoids targeting CB2Rs in dictating the basal expression of these genes.

### CB2R Maintains the Levels of Na^+^/K^+^-ATPase in the Stria Vascularis

**FIGURE 6:**
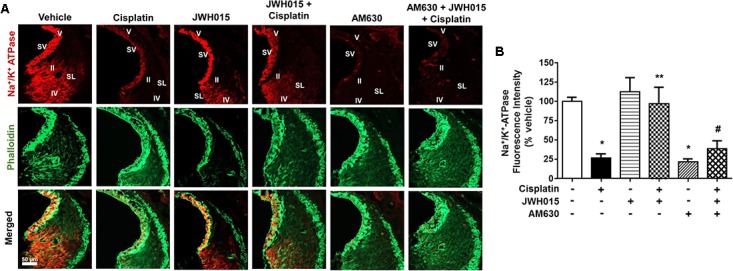
Activation of CB2R by JWH015 mitigates cisplatin-induced loss of Na^+^/K^+^-ATPase-α1 levels in the rat cochlea. **(A)** Mid-modiolar sections of the rat cochlea were co-immunolabeled with Na^+^/K^+^-ATPase-α1 and phalloidin. The sections were imaged using Zeiss LSM800 scanning confocal microscope. Representative images from the base of the cochlea are shown (*n* ≥ 3). **(B)** Bar graph represents % Na^+^/K^+^-ATPase fluorescence intensity ± SEM. Cisplatin decreases expression of Na^+^/K^+^-ATPase-α1 in stria vascularis (SV) and spiral ligament (SL) and this is attenuated by pre-treatment with JWH015. AM630 added alone also reduced Na^+^/K^+^-ATPase immunoreactivity which was similar to cisplatin. ^∗^*p* < 0.05 vs. vehicle; ^∗∗^*p* < 0.05 vs. cisplatin; and ^#^*p* < 0.05 vs. JWH015 + cisplatin (one-way ANOVA).

Na^+^/K^+^-ATPase present in the SV and SL fibrocytes plays a critical role in maintaining endocochlear potential (EP) in the cochlea (van Benthem et al., 1994; Wangemann, 2002). Recently we have shown that cisplatin reduces the levels of this protein in SV and SL (Borse et al., 2017) which might contribute to disruption of EP and hearing loss. In this study, we determined whether activation of CB2R could also attenuate cisplatin-induced loss of Na^+^/K^+^-ATPase using an antibody which labels the α1 subunits of this enzyme. We show that cisplatin reduced the level of Na^+^/K^+^-ATPase in the SV which was abrogated by CB2R activation (**Figures 6A,B**). Cisplatin reduced Na^+^/K^+^-ATPase α1 immunoreactivity to 26.4 ± 5.2% of vehicle-treated controls, which was attenuated in rats pretreated with JWH015 (labeling was reduced to 96.9 ± 21.1% of vehicle-treated controls). No significant change was observed in Na^+^/K^+^-ATPase α1 immunoreactivity in SV from rats treated with JWH015 alone. Surprisingly, AM630 added alone reduced Na^+^/K^+^-ATPase α1 immunoreactivity to an extent which was comparable to cisplatin (21.4 ± 3.6%). In addition, administration of AM630 blocked the increase in Na^+^/K^+^-ATPase α1 immunoreactivity produced by JWH015 in presence of cisplatin (38.4 ± 10%). These data suggest that Na^+^/K^+^-ATPase could serve as another target of cisplatin for inducing hearing loss which is regulated by CB2R activation. Furthermore, the findings with AM630 lend support to the conclusion that the basal expression and/or distribution of this enzyme in the SV is strictly dependent on CB2R.

### Knockdown of CB2R in the Cochlea Exacerbates Cisplatin-Induced Hearing Loss

**FIGURE 7:**
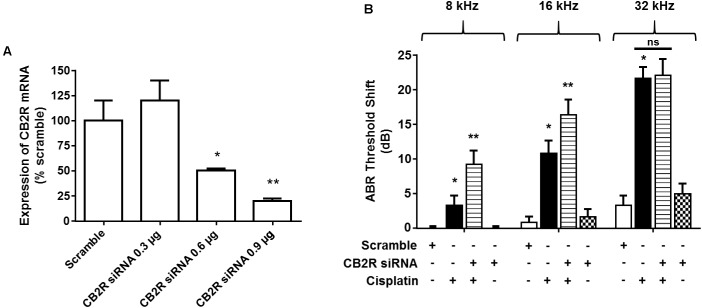
Knock-down of CB2R expression by siRNA increases the sensitivity to cisplatin-induced hearing loss. **(A)** Rats were administered either scramble siRNA (0.9 μg) or increasing doses of CB2R siRNA (0.3, 0.6, and 0.9 μg) by *trans*-tympanic injections. The animals were sacrificed 48 h later and mRNA was assessed for CB2R expression by real-time RT-PCR. CB2R siRNA knocked down CB2R gene expression by ∼50 and ∼80% using 0.6 μg and 0.9 μg, respectively. Data are represented as % expression ± SEM (*n* = 3). ^∗^*p* < 0.05 vs. scramble siRNA and ^∗∗^*p* < 0.05 vs. CB2R siRNA 0.6 μg. **(B)** Baseline ABR was recorded in rats administered with either a scramble siRNA or CB2R siRNA by *trans*-tympanic injections for 48 h. Cisplatin (11 mg/kg, i.p.) was then administered and post-ABR was recorded 72 h later. There was no significant change in ABR threshold shift in the animals treated with scramble siRNA or CB2R siRNA alone. Pre-treatment with CB2 siRNA (0.9 μg) followed by cisplatin significantly potentiated cisplatin-induced hearing loss at 8 and 16 kHz. Data are represented as mean ± SEM. ^∗^*p* < 0.05 vs. scramble siRNA and ^∗∗^*p* < 0.05 vs. cisplatin (*n* = 6, one-way ANOVA).

*Trans*-tympanic administration of CB2R siRNA produced dose-dependent reduction in total cochlear CB2R mRNA within 48 h, as assessed by RT-PCR (**Figure 7A**). Approximately 80% reduction was observed at the highest dose of siRNA (0.9 μg) used. Lower levels of siRNAs were less effective. At 48 h post siRNA administration, animals were administered vehicle or cisplatin (11 mg/kg) by the intraperitoneal route. Post-treatment ABRs were assessed 3 days later at 8, 16, and 32 kHz. As observed previously, cisplatin produced significant ABR threshold shifts which increased with increasing sound frequencies. Rats which were treated with CB2R siRNA, followed by cisplatin, showed significantly increased threshold shifts at 8 and 16 kHz over those observed in the scrambled siRNA plus cisplatin group. No difference was observed at the 32 kHz frequency (**Figure 7B**). These results are consistent with the conclusion that the endocannabinoid/CB2R system in the cochlea can partially counter cisplatin-induced ototoxicity in the 8 and 16 kHz frequency range. The lack of effect at the high frequency (32 kHz) region of the cochlea could reflect greater toxicity in the base of the cochlea where cisplatin’s concentrations are the greatest (Hellberg et al., 2013) and which overwhelms the protection afforded by CB2R.

### JWH015 Does Not Interfere With Cisplatin Anti-cancer Efficacy in Cancer Cells

**FIGURE 8:**
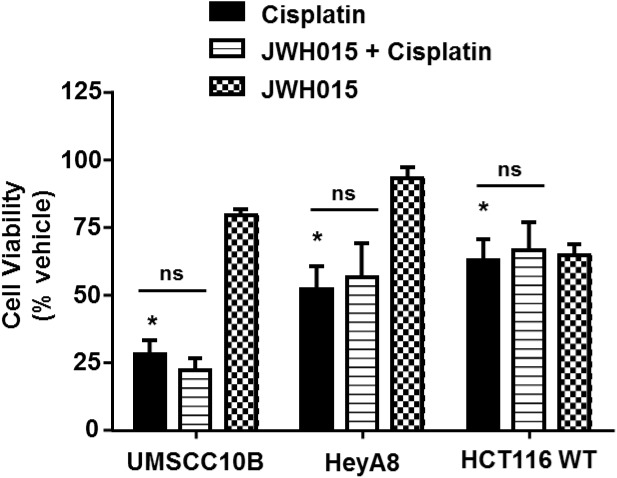
Pre-treatment with JWH015 does not interfere with cisplatin anti-cancer efficacy *in vitro*. Different cancer cells (UMSCC10B, HeyA8, and HCT116 WT) were treated with JWH015 (10 μM) for 30 min followed by cisplatin (10 μM) for 48 h. The cell viability was measured by MTS assay. The percent cell viability is represented in the bar graph as mean ± SEM of at least four independent experiments. ^∗^*p* < 0.05 represent significant difference from vehicle-treated group at 100% (one-way ANOVA).

Previous studies have shown that endogenous and synthetic cannabinoids produce anti-cancer properties. For example, activation of CBRs induce apoptosis in cancer cells by the classical cell death pathways (Olea-Herrero et al., 2009), induce autophagy-mediated cell death (Salazar et al., 2009), arrest the cell cycle and cell division (Laezza et al., 2006) or inhibit neovascularization (Blazquez et al., 2003). We decided to study whether CB2R agonists could interfere with cisplatin chemotherapeutic effects in the event that these agents need to be administered systemically to treat hearing loss. Several cell lines derived from tumors known to be responsive to cisplatin were examined. These include head and neck cancer cells (UMSCC10B), ovarian cancer cells (HeyA8) and colon cancer cells (HCT116 WT). Administration of 10 μM cisplatin to cell cultures produced significant reductions in cell viability which averaged 28.0 ± 5.1%, 52.1 ± 8.6%, and 62.6 ± 7.8% of control cells, respectively, in UMSCC10B, HeyA8, and HCT116 WT cells (**Figure 8**). In cells pre-treated with 10 μM JWH015, the percent cell viability in the JWH-015 + cisplatin group were 22.0 ± 4.5, 56.4 ± 12.5, and 66.4 ± 10.6%, respectively, in UMSCC10B, HeyA8, and HCT116 WT cells. These values were not significantly different from those obtained with cisplatin added alone, suggesting a lack of interference of cisplatin’s anti-cancer response in presence of this CB2R agonist.

## Discussion

The current study demonstrates that CB2Rs are distributed throughout the cochlea, including sensory hair cells, SG neurons, SV and SL. Activation of CB2R by JWH015 protected against cisplatin-induced hearing loss and this protective effect could be completely blocked by AM630, an antagonist of CB2R. Surprisingly, AM630 potentiated the ototoxicity produced by cisplatin, especially at the low frequency range, and produced significant hearing loss when administered alone. These data suggest that activation of CB2Rs (by endocannabinoids) plays a tonic protective role under normal condition and that interference with these receptors or the endogenous cannabinoid system could compromise hearing. In this regard, we observed that knockdown of CB2R in the cochlea by siRNA exacerbated cisplatin-induced hearing loss (at lower frequencies). In addition, this study identified different cochlear targets of the CB2R agonist, JWH015, which could account for its otoprotective actions. These include the OHCs, ribbons synapses and stria vascularis (Na^+^/K^+^-ATPase), regional targets which are shown to express the CB2R subtype.

Systemic administration of cisplatin to the male Wistar rat elevated ABR thresholds, which is especially evident in the basal region of the cochlea. This loss of hearing in the high frequency range was associated with a reduction in OHCs in the basal turn of the cochlea and was attenuated by *trans*-tympanic administration of JWH015, an agonist of the CB2R. JWH015 also protected against hearing loss in the lower frequency range, even though these regions did not experience much discernable damage or loss of OHCs. Thus, other mechanisms could account for the “additional” protection afforded by CB2R in the low frequency range.

Other targets of cisplatin’s action and CB2R-mediated protection include functional ribbon synapses associated with the IHCs. These synapses communicate the original auditory signal generated by the IHCs to SG neurons. Alteration in the number and/or function of these synapses would alter the auditory transmission to SG neurons and central auditory pathways. The reductions in ribbon synapses, or synaptopathy, observed in cochleae obtained from cisplatin-treated rats would therefore contribute to the overall elevations in ABR thresholds which were observed. The basis for this synaptopathy induced by cisplatin which has previously been described (Borse et al., 2017) is not clear, but could result from glutamate excitotoxicity (Puel et al., 1998). Cochlear synaptopathy resulting from noise exposure has been previously described (Liberman, 2016). Previous studies have reported that synapse loss in noise and age-induced hearing loss (Sergeyenko et al., 2013; Espejo-Porras et al., 2015; Liberman and Kujawa, 2017) which was not associated with loss of OHCs or ABR threshold shifts. This type of cochlea synaptopathy is known as “hidden hearing loss” (Liberman and Kujawa, 2017) and is detected by a reduction in wave I supra-threshold amplitude (Sredni et al., 2016; Liberman and Kujawa, 2017). The present study revealed an important “tonic” role of the endocannabinoid/CB2R system to preserve synaptic integrity under normal condition which was unmasked by the application of CB2R antagonist. Since noise-induced temporary threshold shift and resulting synaptopathy is associated with reductions in wave I amplitudes (Liberman, 2016), it is possible that CB2R agonists could similarly protect against this deficit. Whether activation of the CB2R could initiate regeneration of synapses, following trauma, similar to neurotrophin 3 (Suzuki et al., 2016), is not yet known. Unlike noise, cisplatin-induced trauma results in permanent peripheral deficits which are not restored by subsequent administration of protective agents. We observed a significant decrease in wave I amplitudes at 60, 70, 80, and 90 dB in the high frequency region (32 kHz) in both cisplatin and AM630 treatment groups which reflect synapse loss. The mechanism underlying the preservation of synaptic integrity by pretreatment with CB2R agonist is not clear. One possibility is that activation of presynaptic CB2R reduces the release of excessive glutamate and this limits activation of postsynaptic glutamate receptors and damage to the afferent fibers. In support of this hypothesis, we observed co-localization of CB2R and CtBP2 protein in the presynaptic nerve terminal in the mouse cochlea. Previous studies have demonstrated a role of presynaptic CB2R in autaptic hippocampal neurons which mediate presynaptic inhibition of neurotransmitter release (Atwood et al., 2012). In other disease models, upregulation of CB2R has been shown to mediate an “autoprotective” role (Pertwee, 2009). Increased expression of CB2R was observed in neuropathic pain models (Zhang et al., 2003) and in rat sensory neurons during peripheral nerve injury (Wotherspoon et al., 2005).

While CB1Rs are expressed in the cochlea (unpublished data), whether they are expressed in the IHC synapse and to what extent they modulate afferent neurotransmission is unknown. Nevertheless, in the CNS, CB1R plays a major role in regulating presynaptic release of excitatory and inhibitory neurotransmitters, such as GABA and glutamate (Pertwee, 2009).

A significant finding of this study is the observation that perturbation of the endocannabinoid system in the cochlea contributes to hearing loss. We showed that inhibition or knockdown of CB2R resulted in elevations in ABR thresholds. These findings suggest that the endocannabinoid system provides a tonic level of protection to the cochlea which is only revealed upon inhibition of this system with AM630. In addition to the CBRs, we could detect labeling of the endocannabinoid 2-AG synthetic enzyme, diacylglycerol lipase, to similar locations in the cochlea (unpublished data). As such, 2-AG released by cells in the cochlea would be able to activate CB2R on the same or neighboring cells. Such an endogenous system could contribute to cytoprotection under normal physiological activity but could be overwhelmed by ototoxic drugs, such as cisplatin. In such a situation, exogenously administered CB2R agonist could be required to boost the protection afforded by the endocannabinoid system. Another interesting observation is that knockdown of CB2R by siRNA partly mimicked the response to AM630, in that this perturbation sensitized these animals to cisplatin-induced hearing loss at the lower frequencies. These data would support the conclusion that endocannabinoids (probably by acting through CB2R) help to maintain the integrity of IHC ribbon synapse and protect hearing. Another explanation is that in the presence of CB2R blockade the full effects of endocannabinoids on other targets, such as CB1R and TRPV1 channels (Pertwee, 2009) are realized. Accordingly, activation of these targets by endocannabinoids could confer ototoxicity.

The hearing loss produced by AM630 was not associated with loss of OHCs and IHCs, but was more correlated with a loss of IHC ribbon synapses. This would suggest that protecting synaptic integrity and maintenance of EP are more important roles of endocannabinoids than protecting the sensory hair cells. Thus, the hearing loss produced by AM630 is partially different from that produced by cisplatin, as the latter also mediates apoptosis of hair cells. Changes in the EP have been associated with acquired hearing loss including chemotherapeutic drugs, age and noise-induced hearing loss (Schulte and Schmiedt, 1992; Tsukasaki et al., 2000; Hirose and Liberman, 2003). Na^+^/K^+^-ATPase present in the SV plays a critical role in generating high K^+^ concentrations in the endolymph and in maintaining the EP (Hibino et al., 2010). Increased oxidative stress has been shown to decrease the activity of Na^+^/K^+^-ATPase by promoting its proteolytic degradation and endocytosis (Chen et al., 2012). Activation of CB2R protects against cisplatin-induced loss of OHCs in the basal turn of the cochlea. This protection is similar to that observed following activation of the adenosine A_1_ receptor (Kaur et al., 2016), where it is linked to suppression of a NOX3 NADPH oxidase/STAT1 inflammatory pathway. It is believed that inhibition of STAT1 in these OHCs attenuated cisplatin-mediated p53 activation and apoptosis. Inhibition of STAT1 as an otoprotective strategy has been documented in several studies (Schmitt et al., 2009; Mukherjea et al., 2011; Kaur et al., 2016; Borse et al., 2017). We showed that JWH015 suppressed cisplatin-induced STAT1 activation *in vitro*, which could account for its anti-apoptotic role in UB/OC-1 cell cultures (unpublished data). A similar mechanism *in vivo* could account for the preservation of OHCs in the basal turn of the cochlea. In support of this, we show that JWH015 suppressed the induction of a number of STAT1-regulated inflammatory genes, such as *TNF-α*, *COX2*, and *iNOS* in whole cochlear extracts. The expression of *NOX3* has been positively linked to activation of STAT1, as would be expected from an increase in oxidative stress Kaur et al., 2016; Borse et al., 2017).

CB2R plays a major role in regulating the immune system. In addition to suppressing STAT1-dependent inflammatory cytokines and related genes, activation of CB2R suppress the recruitment of inflammatory cells to the site of inflammation. (Klein, 2005). Activation of CB2R also suppress the proliferation and phagocytotic function of macrophages (Ashton and Glass, 2007). CB2R agonist has also been shown to promote apoptosis of immune cells (Lombard et al., 2007). Therefore, yet unexplored actions of JWH015 in this study are its effects on the resident immune cells and the recruitment and apoptosis of circulating immune cells to the cochlea. Such actions would be expected to reduce overall cochlear inflammation and reduce hearing loss. We hope to explore these additional actions of CB2R in the cochlea in the future.

Stimulation of CB2R can exert different effects on cancer cells in a tissue and tumor specific manner (Velasco et al., 2016). Various clinical studies have evaluated the role of cannabinoids for palliative care in cancer patients to alleviate chemotherapy-induced nausea and vomiting (Rock and Parker, 2016) and cancer pain (Johnson et al., 2010). In this study, we show JWH015 does not interfere with cisplatin’s anti-cancer efficacy in head and neck, ovarian and colon cancer cells. These findings need to be further studied in the xenograft mice model and other models before systemic use of JWH015 could be initiated.

In summary, our data support a role of the endocannabinoid/CB2R system in maintaining normal hearing in the rat cochlea. Protection stems from endocannabinoid/CB2R interaction to maintain the integrity of OHCs, IHC ribbon synapses and Na^+^/K^+^-ATPases in the SV. The endocannabinoid/CB2R system is not fully able to protect against cisplatin ototoxicity and requires addition of exogenous CB2R agonist. This agonist protects against the loss of OHCs and ribbon synapses. *Trans*-tympanic administration of CB2R agonist provides effective protection against cisplatin-induced hearing loss and should produce limited side effects of this drug. Based on these results, we would recommend the use of localized administration of CB2R agonists for the treatment of cisplatin-induced hearing loss.

## Author Contributions

SG and VR developed the idea for the research mentioned in this article. SG, SS, and VR wrote the manuscript and edited the figures. KS, SG, SS, AD, and VB performed the experiments while SS, VB, and AD helped with data analysis. DM and LR critiqued and revised the manuscript.

## Conflict of Interest Statement

The authors declare that the research was conducted in the absence of any commercial or financial relationships that could be construed as a potential conflict of interest.

## Supplementary Material

FIGURE S1 Characterization of the CB2R. **(A)** Specificity of CB2R immunolabeling was confirmed in mid-modiolar sections using a blocking peptide for the CB2R protein which revealed an absence of labeled proteins. **(B)** UB/OC1 cells were transiently transfected with a plasmid vector expressing CB2R. Cells were fixed in 4% paraformaldehyde, washed with PBS and then immunolabeled for CB2R (green). Labeling in the untransfected cells was confined to the plasma membrane and was substantially increased following transient transfection of CB2R.

FIGURE S2 Administration of AM630 alone does not reduce OHC number and did not alter the effect of cisplatin. The animals were sacrificed at the end point and cochleae were collected, fixed by 4% PFA in PBS followed by decalcification in 100 mM EDTA for 3 weeks. The base of the cochlea were dissected out and stained with hair cell marker, myosin VIIa, to determine the hair cell loss. Representative images are shown. Scale bar is 20 μm.

TABLE S1 Description of antibodies used.
