# Supplementary figures and images for: The Endocannabinoid/Cannabinoid Receptor 2 System Protects Against Cisplatin-Induced Hearing Loss
Source: Front Cell Neurosci. 2018 Aug 21;12:271. doi: 10.3389/fncel.2018.00271 (PMC6110918; doi:10.3389/fncel.2018.00271)

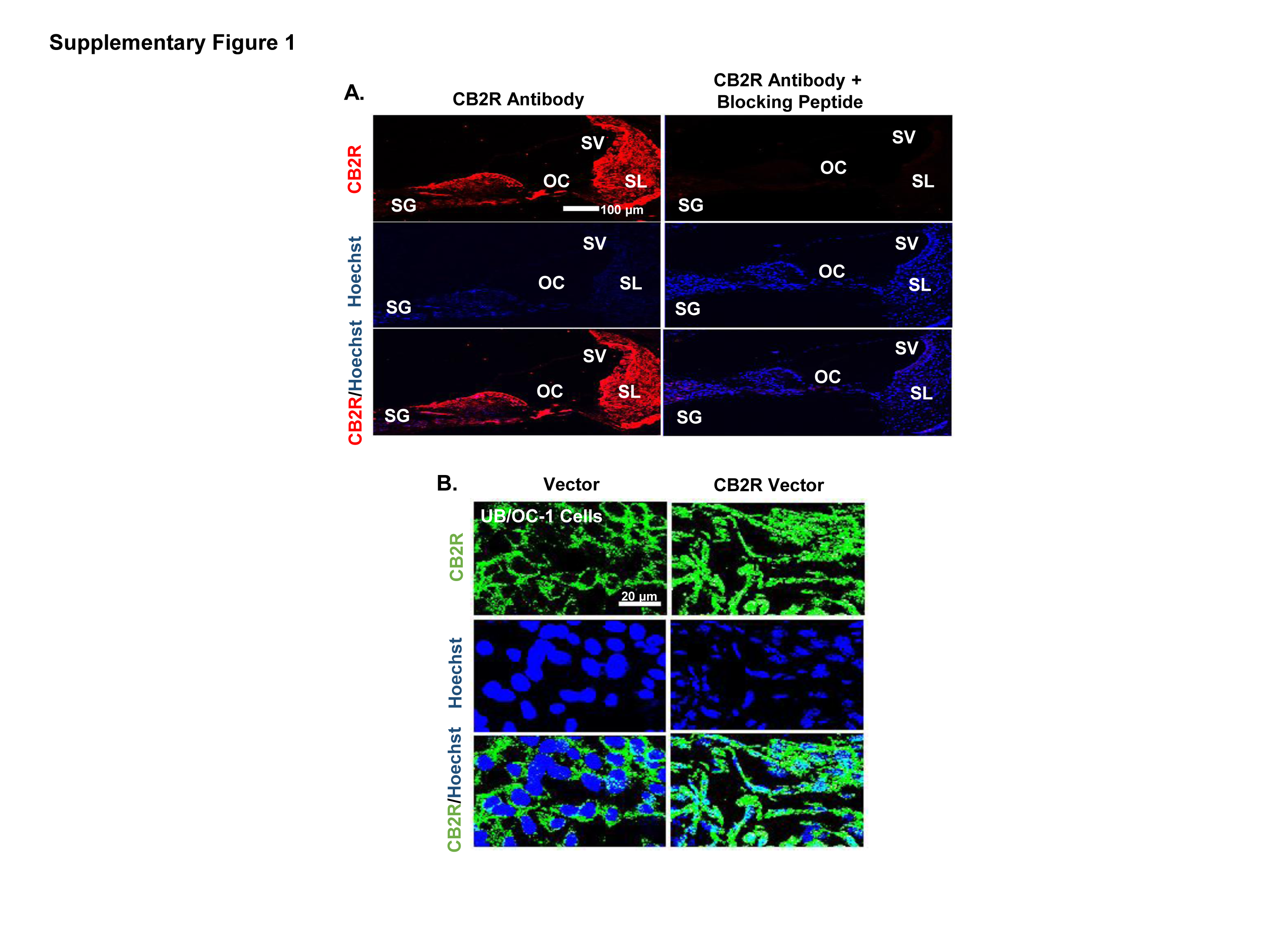

Supplement: FIGURE S1 — Characterization of the CB2R. (A) Specificity of CB2R immunolabeling was confirmed in mid-modiolar sections using a blocking peptide for the CB2R protein which revealed an absence of labeled proteins. (B) UB/OC1 cells were transiently transfected with a plasmid vector expressing CB2R. Cells were fixed in 4% paraformaldehyde, washed with PBS and then immunolabeled for CB2R (green). Labeling in the untransfected cells was confined to the plasma membrane and was substantially increased following transient transfection of CB2R. [file Image_1.TIF]

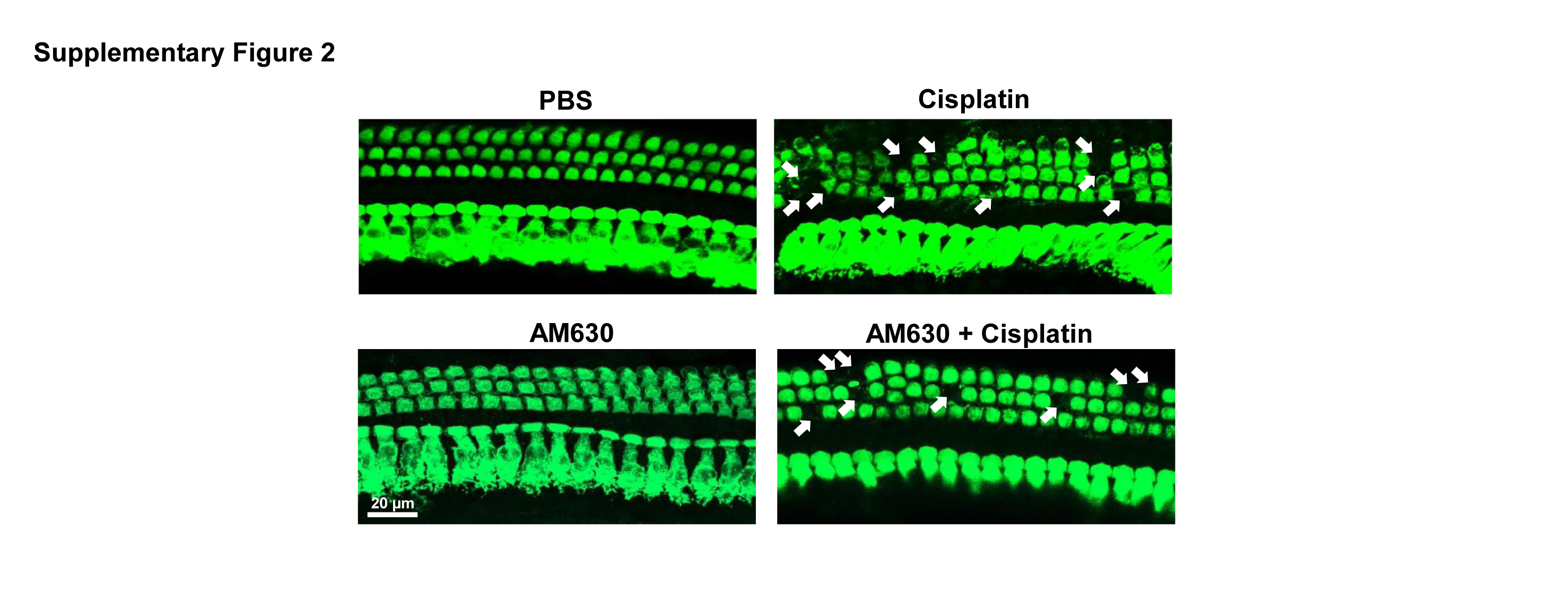

Supplement: FIGURE S2 — Administration of AM630 alone does not reduce OHC number and did not alter the effect of cisplatin. The animals were sacrificed at the end point and cochleae were collected, fixed by 4% PFA in PBS followed by decalcification in 100 mM EDTA for 3 weeks. The base of the cochlea were dissected out and stained with hair cell marker, myosin VIIa, to determine the hair cell loss. Representative images are shown. Scale bar is 20 μm. [file Image_2.TIF]
