# Supplementary material for: The Endocannabinoid/Cannabinoid Receptor 2 System Protects Against Cisplatin-Induced Hearing Loss
Source: Front Cell Neurosci. 2018 Aug 21;12:271. doi: 10.3389/fncel.2018.00271 (PMC6110918; doi:10.3389/fncel.2018.00271)
Supplement: TABLE S1 — Description of antibodies used. [file Data_Sheet_1.PDF]

Table 1: Description of antibodies used.

Name of the Antibody	Species	Species of primary Antibody	Cross-reactivity	Manufacturer	Catalog number	Type of the Antibody	Dilution
Cannabinoid receptor II	Rat	Rabbit	Mouse, Human	Abcam	Ab45942	Polyclonal	1 to 200
Glutamate Receptor 2	Rat	Mouse	Mouse, Monkey, Canine	Millipore	MAB397	Monoclonal IgG2a	1 to 100
Tuj1	Rat	Mouse	Human, Rat, Mouse	Covance	MMS-435P	Monoclonal IgG 2a	1 to 500
Anti-CtBP2	Mouse	Mouse	Human, Rat, Dog	BD Transduction Laboratories	612044	Monoclonal IgG1	1 to 200
Na+/K+-ATPase α1	Rabbit	Mouse	Human	Santa Cruz	21712	Monoclonal IgG1	1 to 50
Myosin-VIIa	Human	Rabbit	Mouse, Rat, Pig, Avian, Amphibian	Proteus Biosciences	25-6790	Polyclonal	1 to 200
Myosin-VIIa	Human	Mouse	Chicken, Mouse, Rat, Zebrafish	Developmental Studies Hybridoma Bank	138-1	Monoclonal IgG1	1 to 50
